# *Heteropodalebar* sp. nov.: a new species from the highlands in Pahang State, Malaysia (Sparassidae, Heteropodinae) with a distinct sexual colour dimorphism

**DOI:** 10.3897/BDJ.12.e125745

**Published:** 2024-06-04

**Authors:** Wenqin Chen, Peter Jäger, Yang Zhu, Long Yu, He Zhang

**Affiliations:** 1 Hubei Key Laboratory of Regional Development and Environmental Response, Faculty of Resources and Environmental Science, Hubei University, Wuhan 430062, Hubei, China Hubei Key Laboratory of Regional Development and Environmental Response, Faculty of Resources and Environmental Science, Hubei University Wuhan 430062, Hubei China; 2 Arachnology, Senckenberg Research Institute, Mertonstraße 17–21, 60325 Frankfurt am Main, Germany Arachnology, Senckenberg Research Institute Mertonstraße 17–21, 60325 Frankfurt am Main Germany; 3 The State Key Laboratory of Biocatalysis and Enzyme Engineering of China, School of Life Sciences, Hubei University, Wuhan 430062, Hubei, China The State Key Laboratory of Biocatalysis and Enzyme Engineering of China, School of Life Sciences, Hubei University Wuhan 430062, Hubei China

**Keywords:** high diversity, Heteropodinae, sexual colour dimorphism, taxonomy

## Abstract

**Background:**

The genus *Heteropoda* Latreille, 1804, is ranked as the second within the family Sparassidae Bertkau, 1872. Up to now, sixteen species of this genus have been described from Malaysia.

**New information:**

A new species of this genus in the highlands of Pahang State, Malaysia is described under the name of *H.lebar* **sp. nov.**. Individuals of the new species live in primary forests on forest floor, active in the night on the leaf litter.

## Introduction

*Heteropoda* Latreille, 1804 (Latreille 1804), is a large genus within the subfamily Heteropodinae (Thorell 1897), with 188 nominal species and mainly distributed in Asia and Australia (WSC 2024). Revisionary work has been done so far mainly for Southeast Asia and Australia (e.g Davies (1994), Jäger (2002), Jäger (2005), Jäger (2008), Jäger (2014)). The majority of the diversity of the genus is still unrevised or undiscovered.

New material recorded from Pahang State, Malaysia, revealed a species new to science with a distinct sexual colour dimorphism. It is diagnosed, described and illustrated and a distribution map is provided.

## Materials and methods

All spiders are preserved in 70% denatured ethanol. Observations and drawings were made using a Leica MZ 16 stereomicroscope and a Leica DLMS compound microscope, each with a camera lucida attachment. Photographs of live and preserved specimens were taken with a Canon EOS R and a Canon 100 mm macro lens in combination with a Canon MR 14EX ringlite. Photographs of copulatory organs were taken with a Leica DMC4500 digital camera attached to a Leica M205 C digital microscope.

Prosoma length/width is the length/width of the dorsal shield of the prosoma, opisthosoma length/width is the length excluding petiolus and spinnerets. Eye distances were measured in orthogonal views. Leg formula, leg spination pattern and size classes follow Jäger (2001). Palp and leg lengths are given as: total (femur, patella, tibia, metatarsus, tarsus). The arising points of appendages of the male bulb are given in clock-position of the left palp in ventral view. Colouration is described from specimens in ethanol and live specimens. All measurements are given in millimetres. Data in square brackets were retrieved subsequently. Elevation of localities are given in metres (m). The map was produced using ArcMap version 10.8.1.

Abbreviations used in the text and figures: AB—anterior bands, ALE—anterior lateral eyes, AME—anterior median eyes, C—conductor, dRTA—dorsal branch of RTA, DS—dorsal shield of prosoma, E—embolus, EF—epigynal field, FD—fertilisation duct, Fe—femur/femora, FW—first winding of internal duct system, GP—glandular pores, LL—lateral lobes, MS—median septum, Mt—metatarsus/metatarsi, OL—opisthosoma length, OS—opisthosoma, OW—opisthosoma width, Pa—patella/patellae, PL—prosoma length, PLE—posterior lateral eyes, PME—posterior median eyes, PP—posterior part of internal duct system, PW—prosoma width, RTA—retrolateral tibial apophysis, S—spermophor, SH—spermathecal head, SO—spermophor opening, SP—septal pocket, SS—slit sensillum, Ta—tarsus/tarsi, Ti—tibia/tibiae, TL—total length, vRTA—ventral branch of RTA.

Museum collections (with curators): CBEE—Centre for Behavioural Ecology and Evolution, College of Life Sciences, Hubei University, Wuhan, China (Yang Zhu). LKCNHM—Lee Kong Chian Natural History Museum, Singapore (Wendy Wang). SMF—Senckenberg Research Institute, Frankfurt am Main, Germany (J. Grüger, P. Jäger).

## Taxon treatments

### Heteropoda lebar sp. nov.

5785E973-6DE1-5803-B048-BB2506123969

B7980E1D-13EF-4AC6-8D58-8CA45496CCA3

#### Materials

- **Type status:** Holotype. **Occurrence:** recordedBy: P. Jäger; individualCount: 1; sex: male; lifeStage: adult; preparations: whole animal (ETOH); occurrenceID: 0818B12E-DE6B-57AD-B0E9-0A33EAD5F315; **Taxon:** scientificName: *Heteropodalebar* sp. nov.; order: Araneae; family: Sparassidae; genus: Heteropoda; **Location:** country: Malaysia; stateProvince: Pahang; county: Bukit Fraser; locality: South Fraser’s Hill; verbatimElevation: 1260 m; verbatimLatitude: 3°42'27.2"N; verbatimLongitude: 101°44'16.0"E; verbatimSRS: WGS84; **Identification:** identifiedBy: P. Jäger; **Event:** samplingProtocol: by hand; year: 2013; month: June; day: 18; eventRemarks: disturbed primary forest, along road, at night; **Record Level:** institutionCode: SMF
- **Type status:** Paratype. **Occurrence:** recordedBy: P. Jäger; individualCount: 1; sex: female; lifeStage: adult; preparations: whole animal (ETOH); occurrenceID: 290FCAA1-4EF3-59F6-9594-BFAA386B720E; **Taxon:** scientificName: *Heteropodalebar* sp. nov.; order: Araneae; family: Sparassidae; genus: Heteropoda; **Location:** country: Malaysia; stateProvince: Pahang; county: Bukit Fraser; locality: South Fraser’s Hill; verbatimElevation: 1260 m; verbatimLatitude: 3°42'27.2"N; verbatimLongitude: 101°44'16.0"E; verbatimSRS: WGS84; **Identification:** identifiedBy: P. Jäger; **Event:** samplingProtocol: by hand; year: 2013; month: June; day: 18; eventRemarks: disturbed primary forest, along road, at night; **Record Level:** institutionCode: SMF
- **Type status:** Paratype. **Occurrence:** recordedBy: P. Jäger; individualCount: 2; sex: males; lifeStage: adult; preparations: whole animal (ETOH); occurrenceID: 7ED0412A-D32F-5E3B-B0FE-7D6487599B98; **Taxon:** scientificName: *Heteropodalebar* sp. nov.; order: Araneae; family: Sparassidae; genus: Heteropoda; **Location:** country: Malaysia; stateProvince: Pahang; county: Bukit Fraser; locality: South Fraser’s Hill; verbatimElevation: 1260 m; verbatimLatitude: 3°42'27.2"N; verbatimLongitude: 101°44'16.0"E; verbatimSRS: WGS84; **Identification:** identifiedBy: P. Jäger; **Event:** samplingProtocol: by hand; year: 2013; month: June; day: 18; eventRemarks: disturbed primary forest, along road, at night; **Record Level:** institutionCode: LKCNHM
- **Type status:** Paratype. **Occurrence:** recordedBy: P. Jäger; individualCount: 1; sex: male; lifeStage: adult; preparations: whole animal (ETOH); occurrenceID: BF4BC5BC-D3FA-55CC-89B3-CA32B794910C; **Taxon:** scientificName: *Heteropodalebar* sp. nov.; order: Araneae; family: Sparassidae; genus: Heteropoda; **Location:** country: Malaysia; stateProvince: Pahang; county: Bukit Fraser; locality: Telecom look; verbatimElevation: 1300 m; verbatimLatitude: 3°43'6.3"N; verbatimLongitude: 101°45'9.86"E; verbatimSRS: WGS84; **Identification:** identifiedBy: P. Jäger; **Event:** samplingProtocol: by hand; year: 2013; month: June; day: 16; eventRemarks: disturbed primary forest, along road, at night; **Record Level:** institutionCode: SMF
- **Type status:** Paratype. **Occurrence:** recordedBy: P. Jäger; individualCount: 1; sex: male; lifeStage: adult; preparations: whole animal (ETOH); occurrenceID: DC40FFD3-1C51-5926-A461-71B4DF5F88B4; **Taxon:** scientificName: *Heteropodalebar* sp. nov.; order: Araneae; family: Sparassidae; genus: Heteropoda; **Location:** country: Malaysia; stateProvince: Pahang; county: Bukit Fraser; locality: Telecom look; verbatimElevation: 1300 m; verbatimLatitude: 3°43'6.3"N; verbatimLongitude: 101°45'9.86"E; verbatimSRS: WGS84; **Identification:** identifiedBy: P. Jäger; **Event:** samplingProtocol: by hand; year: 2013; month: June; day: 16; eventRemarks: disturbed primary forest, along road, at night; **Record Level:** institutionCode: LKCNHM
- **Type status:** Paratype. **Occurrence:** recordedBy: P. Jäger; individualCount: 1; sex: female; lifeStage: adult; preparations: whole animal (ETOH); occurrenceID: 8ED01EBB-1164-5993-890B-5F00240B55EE; **Taxon:** scientificName: *Heteropodalebar* sp. nov.; order: Araneae; family: Sparassidae; genus: Heteropoda; **Location:** country: Malaysia; stateProvince: Pahang; county: Bukit Fraser; locality: Telecom look; verbatimElevation: 1300 m; verbatimLatitude: 3°43'6.3"N; verbatimLongitude: 101°45'9.86"E; verbatimSRS: WGS84; **Identification:** identifiedBy: P. Jäger; **Event:** samplingProtocol: by hand; year: 2013; month: June; day: 16; eventRemarks: disturbed primary forest, along road, at night; **Record Level:** institutionCode: SMF
- **Type status:** Paratype. **Occurrence:** recordedBy: P. Jäger & T. Laufs; individualCount: 1; sex: male; lifeStage: adult; preparations: whole animal (ETOH); occurrenceID: 1190491B-2C45-5F3D-AF30-A10FE194EA65; **Taxon:** scientificName: *Heteropodalebar* sp. nov.; order: Araneae; family: Sparassidae; genus: Heteropoda; **Location:** country: Malaysia; stateProvince: Pahang; county: Bukit Fraser; locality: Telecom look; verbatimElevation: 1300 m; verbatimLatitude: 3°43'6.3"N; verbatimLongitude: 101°45'9.86"E; verbatimSRS: WGS84; **Identification:** identifiedBy: P. Jäger; **Event:** samplingProtocol: by hand; year: 2015; month: February; day: 14; eventRemarks: disturbed secondary forest, along road, at night; **Record Level:** institutionCode: SMF
- **Type status:** Paratype. **Occurrence:** recordedBy: P. Jäger & T. Laufs; individualCount: 1; sex: female; lifeStage: adult; preparations: whole animal (ETOH); occurrenceID: 1190491B-2C45-5F3D-AF30-A10FE194EA65; **Taxon:** scientificName: *Heteropodalebar* sp. nov.; order: Araneae; family: Sparassidae; genus: Heteropoda; **Location:** country: Malaysia; stateProvince: Pahang; county: Bukit Fraser; locality: Telecom look; verbatimElevation: 1300 m; verbatimLatitude: 3°43'6.3"N; verbatimLongitude: 101°45'9.86"E; verbatimSRS: WGS84; **Identification:** identifiedBy: P. Jäger; **Event:** samplingProtocol: by hand; year: 2015; month: February; day: 14; eventRemarks: disturbed secondary forest, along road, at night; **Record Level:** institutionCode: SMF
- **Type status:** Paratype. **Occurrence:** recordedBy: P. Jäger & T. Laufs; individualCount: 1; sex: male; lifeStage: adult; preparations: whole animal (ETOH); occurrenceID: 0BFAC03B-8FC5-5A9E-88B7-DBC5CD5525B9; **Taxon:** scientificName: *Heteropodalebar* sp. nov.; order: Araneae; family: Sparassidae; genus: Heteropoda; **Location:** country: Malaysia; stateProvince: Pahang; county: Bukit Fraser; locality: Telecom look; verbatimElevation: 1186 m; locationRemarks: between those coordinates given and pine tree trail; verbatimLatitude: 3°42'49.99"N; verbatimLongitude: 101°43'8.95"E; verbatimSRS: WGS84; **Identification:** identifiedBy: P. Jäger; **Event:** samplingProtocol: by hand; year: 2015; month: February; day: 21; eventRemarks: disturbed primary forest, along trail, at night; **Record Level:** institutionCode: LKCNHM
- **Type status:** Paratype. **Occurrence:** recordedBy: P. Jäger & T. Laufs; individualCount: 1; sex: male; lifeStage: adult; preparations: whole animal (ETOH); occurrenceID: D1D48B01-3165-56A5-96D3-80DA0CAA3243; **Taxon:** scientificName: *Heteropodalebar* sp. nov.; order: Araneae; family: Sparassidae; genus: Heteropoda; **Location:** country: Malaysia; stateProvince: Pahang; county: Bukit Fraser; locality: Telecom look; verbatimElevation: 1186 m; locationRemarks: between those coordinates given and pine tree trail; verbatimLatitude: 3°42'49.99"N; verbatimLongitude: 101°43'8.95"E; verbatimSRS: WGS84; **Identification:** identifiedBy: P. Jäger; **Event:** samplingProtocol: by hand; year: 2015; month: February; day: 21; eventRemarks: disturbed primary forest, along trail, at night; **Record Level:** institutionCode: SMF
- **Type status:** Paratype. **Occurrence:** recordedBy: P. Jäger & T. Laufs; individualCount: 2; sex: females; lifeStage: adult; preparations: whole animal (ETOH); occurrenceID: 9677CED8-B6C3-5004-9612-605FC060F0A8; **Taxon:** scientificName: *Heteropodalebar* sp. nov.; order: Araneae; family: Sparassidae; genus: Heteropoda; **Location:** country: Malaysia; stateProvince: Pahang; county: Bukit Fraser; locality: Hemmant trail, Lady Guillemard road; verbatimElevation: 1275 m; verbatimLatitude: 3°42'55.85"N; verbatimLongitude: 101°44'19.15"E; verbatimSRS: WGS84; **Identification:** identifiedBy: P. Jäger; **Event:** samplingProtocol: by hand; year: 2015; month: February; day: 15; eventRemarks: disturbed secondary forest, at night; **Record Level:** institutionCode: SMF
- **Type status:** Paratype. **Occurrence:** recordedBy: P. Jäger & T. Laufs; individualCount: 1; sex: male; lifeStage: adult; preparations: whole animal (ETOH); occurrenceID: E1062C5C-8AB7-5D1F-9A9B-5CBC336A4B80; **Taxon:** scientificName: *Heteropodalebar* sp. nov.; order: Araneae; family: Sparassidae; genus: Heteropoda; **Location:** country: Malaysia; stateProvince: Pahang; county: Bukit Fraser; locality: Jeriau waterfall; verbatimElevation: 1040 m; verbatimLatitude: 3°43'26.07"N; verbatimLongitude: 101°42'36.22"E; verbatimSRS: WGS84; **Identification:** identifiedBy: P. Jäger; **Event:** samplingProtocol: by hand; year: 2015; month: February; day: 13; eventRemarks: disturbed primary forest, along trail, at night; **Record Level:** institutionCode: SMF
- **Type status:** Paratype. **Occurrence:** recordedBy: P. Jäger; individualCount: 1; sex: male; lifeStage: adult; preparations: whole animal (ETOH); occurrenceID: D56887E5-575F-5EE9-A37A-765590070573; **Taxon:** scientificName: *Heteropodalebar* sp. nov.; order: Araneae; family: Sparassidae; genus: Heteropoda; **Location:** country: Malaysia; stateProvince: Pahang; county: Bukit Fraser; locality: Mager and Abu Suradi trail; verbatimElevation: 1250 m; verbatimLatitude: 3°42'35.13"N; verbatimLongitude: 101°44'0.96"E; verbatimSRS: WGS84; **Identification:** identifiedBy: P. Jäger; **Event:** samplingProtocol: by hand; year: 2013; month: June; day: 18; eventRemarks: disturbed primary forest, at night; **Record Level:** institutionCode: SMF
- **Type status:** Paratype. **Occurrence:** recordedBy: P. Jäger & T. Laufs; individualCount: 2; sex: females; lifeStage: adult; preparations: whole animal (ETOH); occurrenceID: E497F9AF-0230-58CC-8DBD-1DD7D4A72C6E; **Taxon:** scientificName: *Heteropodalebar* sp. nov.; order: Araneae; family: Sparassidae; genus: Heteropoda; **Location:** country: Malaysia; stateProvince: Pahang; county: Bukit Fraser; locality: Mager and Abu Suradi trail; verbatimElevation: 1250 m; verbatimLatitude: 3°42'35.13"N; verbatimLongitude: 101°44'0.96"E; verbatimSRS: WGS84; **Identification:** identifiedBy: P. Jäger; **Event:** samplingProtocol: by hand; year: 2015; month: February; day: 10; eventRemarks: disturbed primary forest, at night; **Record Level:** institutionCode: LKCNHM
- **Type status:** Paratype. **Occurrence:** recordedBy: P. Jäger & T. Laufs; individualCount: 2; sex: females; lifeStage: adult; preparations: whole animal (ETOH); occurrenceID: 26E7E9BC-2D14-5AC8-854C-CE3D8C369C45; **Taxon:** scientificName: *Heteropodalebar* sp. nov.; order: Araneae; family: Sparassidae; genus: Heteropoda; **Location:** country: Malaysia; stateProvince: Pahang; county: Bukit Fraser; locality: Peninjan road, close to Tanglin; verbatimElevation: 1237 m; verbatimLatitude: 3°42'51.42"N; verbatimLongitude: 101°44'46.0"E; verbatimSRS: WGS84; **Identification:** identifiedBy: P. Jäger; **Event:** samplingProtocol: by hand; year: 2015; month: February; day: 11; eventRemarks: disturbed secondary forest, embankments along road, at night; **Record Level:** institutionCode: SMF
- **Type status:** Paratype. **Occurrence:** recordedBy: L. Yu; individualCount: 2; sex: 1 male, 1 female; lifeStage: adult; preparations: whole animal (ETOH); occurrenceID: 1ADFDFAB-F949-5C47-8D14-7CE0699ACEDF; **Taxon:** scientificName: *Heteropodalebar* sp. nov.; order: Araneae; family: Sparassidae; genus: Heteropoda; **Location:** country: Malaysia; stateProvince: Pahang; locality: along Road 59, 5 km to Tanah Rata, Cameron Highlands; verbatimElevation: 1399 m; verbatimLatitude: 4°28'3.03"N; verbatimLongitude: 101°22'4.79"E; verbatimSRS: WGS84; **Identification:** identifiedBy: H. Zhang; **Event:** samplingProtocol: by hand; year: 2024; month: January; day: 15; eventRemarks: at night; **Record Level:** institutionCode: CBEE

#### Description

**Male** (holotype): **Measurements**: TL 17.1, PL 8.6, PW 7.7, AW 3.8, OL 8.5, OW 4.8. **Eyes**: AME 0.40, ALE 0.73, PME 0.60, PLE 0.75, AME-AME 0.21, AME-ALE 0.05, PME-PME 0.31, PME-PLE 0.50, AME-PME 0.46, ALE-PLE 0.49, CH AME 0.95, CH ALE 0.60. **Spination**: Pp 131, 101, 2121; Fe I–III 323, IV 33(4)1; Pa I–III 101, IV 10(1)1; Ti I–IV 2226; Mt I–II 1014, III 2014, IV 303(4)6. Mt I–III with dense scopulae along entire length, IV with distal and proximal field and few rows of stronger setae along entire length. **Measurements of palps and legs**: Palp 13.5 (4.4, 2.1, 3.1, -, 3.9); I 48.0 (12.8, 4.8, 14.0, 12.8, 3.6); II 52.6 (14.4., 5.1, 15.4, 14.0, 2.7); III 39.9 (11.5, 4.2, 11.5, 10.0, 2.7); IV 44.1 (12.3, 4.0, 12.1, 12.6, 3.1). Leg formula: II-I-IV-III. Chelicerae with 3 promarginal and 4 retromarginal teeth, ca. 85 denticles in dense patch close to promarginal teeth and one escort seta.

**Figure 1.**
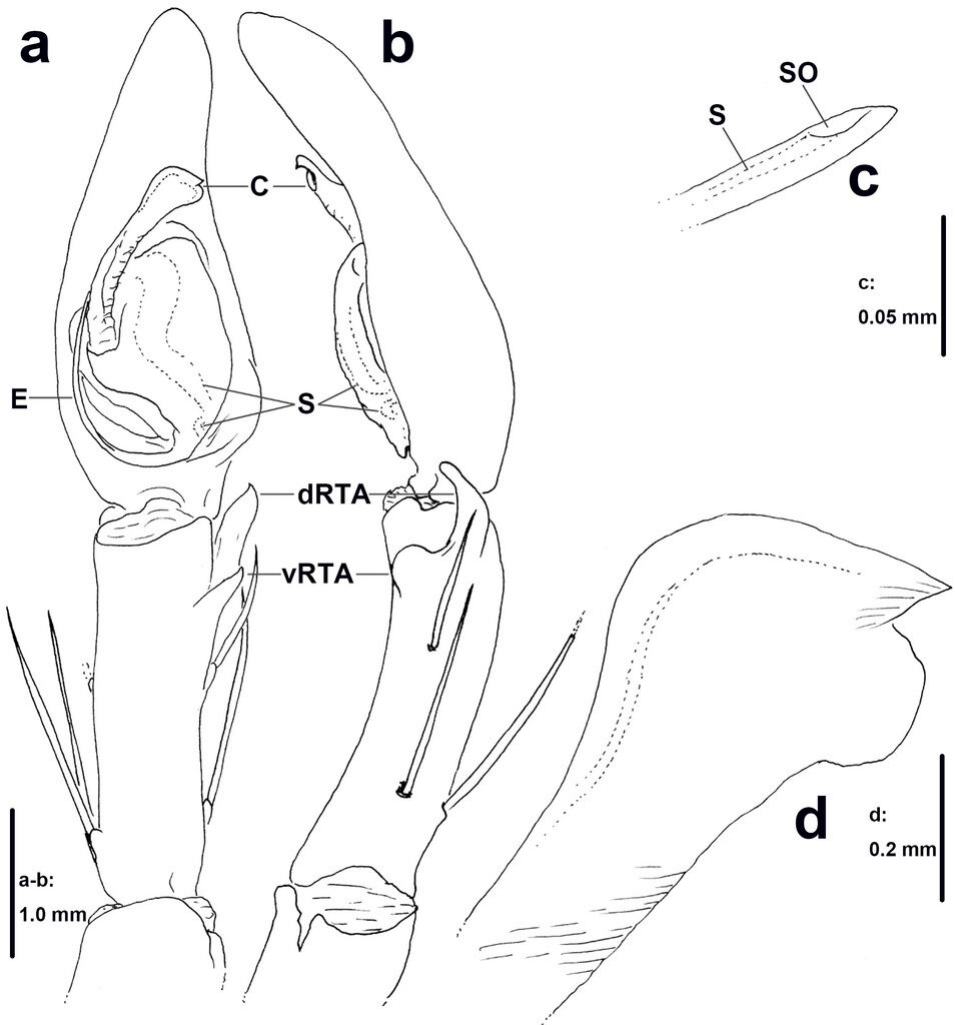
*Heteropodalebar* **sp. nov.**, holotype male from Bukit Fraser, Pahang State, Malaysia, left palp (**a** ventral, **b** retrolateral, **c** embolus tip, ventral, **d** conductor, ventral).

**Palp** (Figs 1, 4a–c). As in diagnosis. RTA arising distally to sub-distally from Ti, vRTA in retrolateral view with rounded hump, dRTA with broader basal part and finger-shaped apical part, in ventral view with short acuminate tip. Cymbium longer than Ti, with indistinct small, rounded retro-proximal swelling. Spermophor slightly S-shaped in ventral view, with distinct loop proximally before entering embolus. Embolus arising from tegulum in 5-o’clock-position, narrow, its tip not easily visible from the ventral view, spermophor opening situated sub-apically. Conductor arising from tegulum in 9-o’clock-position, bluntly widened apically.

**Colouration** (Fig. 5a–b). Yellowish-brown with vivid pattern consisting of dark setae. DS with posterior margin dark, anterior to the typical light crescent and dark area posterior to fovea, fovea with light setae, rest with dark patches, especially with pair of dark elongate patches between eyes and fovea; light patch in front of AME. Chelicerae reddish-brown with three dark longitudinal stripes each. Sternum, ventral coxae, gnathocoxae and labium light yellowish-brown without pattern. Palps and legs ventrally yellowish-brown without pattern, femora dorsally with dark, distinct patches. OS dorsally with dark patches and dark W-shaped pattern in posterior half, heart region lighter; laterally spotted; ventrally yellowish-brown without pattern. Live spiders with a much more contrasting pattern of yellow-brown and dark-brown to black on DS, appendages and dorsal opisthosoma (Fig. 6a).

**Female** (paratype syntopic with holotype): **Measurements**: TL 27.8, PL 11.9, PW 9.8, AW 5.5, OL 15.9, OW 10.7. **Eyes**: AME 0.50, ALE 0.85, PME 0.70, PLE 0.82, AME-AME 0.32, AME-ALE 0.11, PME-PME 0.47, PME-PLE 0.81, AME-PME 0.75, ALE-PLE 0.77, CH AME 1.33, CH ALE 0.81. **Spination**: Pp 131, 101, 2121, 1014; Fe I–III 323, IV 331; Pa I–III 101, IV 001; Ti I–IIII 2026, IV 2126; Mt I–II 1014, III 2014, IV 3036. Mt I–III with dense scopulae along entire length, IV with distal and proximal field and few rows of stronger setae along entire length. **Measurements of palps and legs**: Palp 16.9 (5.1, 2.5, 4.0, -, 5.3); I 44.4 (12.5, 5.5, 12.4, 10.9, 3.1); II 47.8 (13.7, 5.8, 13.5, 11.6, 3.3); III 39.7 (11.6, 4.9, 11.0, 9.3, 2.9); IV 44.9 (13.0, 4.7, 12.0, 11.7, 3.5). Leg formula: II-IV-I-III. Chelicerae with 3 promarginal and 4 retromarginal teeth, 80–90 denticles in dense patch close to promarginal teeth, 8 denticles in proximal half and one escort seta.

**Figure 2.**
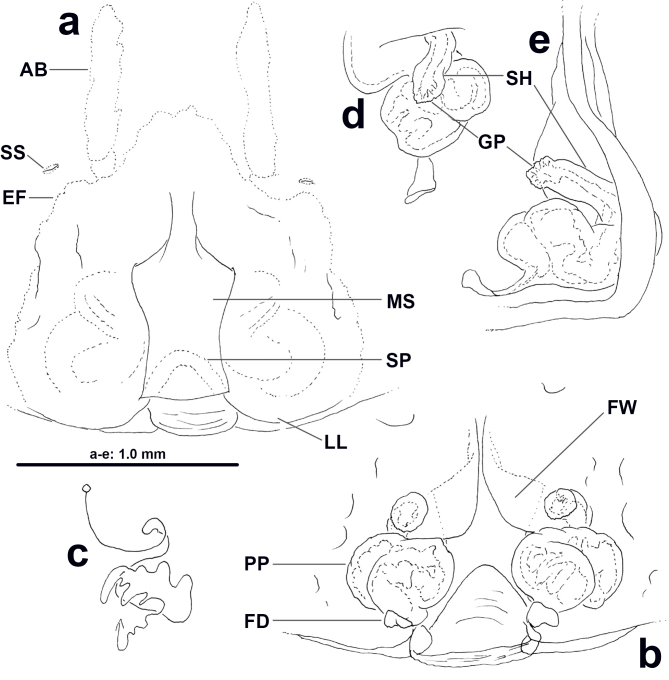
*Heteropodalebar* **sp. nov.**, female paratype from Bukit Fraser, Pahang State, Malaysia. **a** Epigyne, ventral; **b, d–e** Vulva (**b** dorsal, **d** anterior, **e** lateral); **c** Schematic course of internal duct system, dorsal.

**Copulatory organ** (Fig. 2). As in diagnosis. Epigynal field only slightly wider than long, anterior bands distinct, fused, with one slit sensillum on each side close to field. Median septum distinctly longer than wide, with anterior part narrow, septal pocket distinctly developed. First winding wide, ventro-laterad; glandular appendages connected with posterior part by an S-shaped duct, visible in ventral view; posterior part of internal duct system consisting of various bulged parts; fertilisation ducts postero-ventrad.

**Colouration** (Fig. 5c–d). As in male, but generally darker. DS with dense cover of dark setae, light patches visible through this cover. Legs dorsally uniformly covered with dark setae. OS almost uniformly brown, dorsally darker with dense cover of dark setae and light patches, the latter larger in anterior and smaller in posterior half. Live spiders almost uniformly dark brown to black with iridescent purplish shimmer on femora; DS with typical light, almost white crescent submarginally on posterior DS and with many small light spots on legs and OS (Fig. 6b).

**Figure 3.**
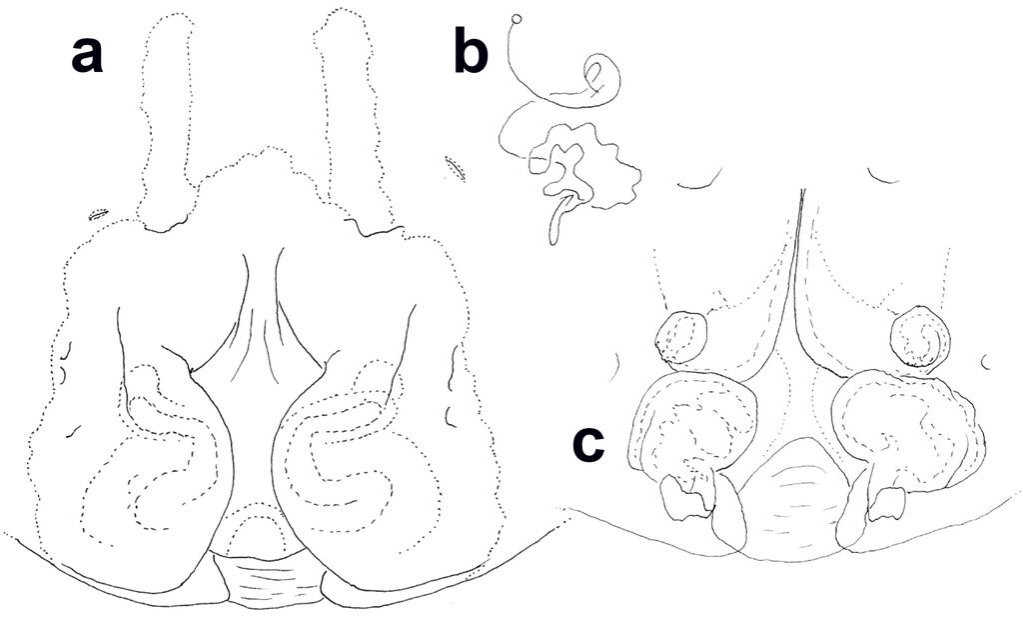
*Heteropodalebar* **sp. nov.**, female paratype from Bukit Fraser, Pahang State, Malaysia. **a** Epigyne, ventral; **b** Schematic course of internal duct system, dorsal; **c** Vulva, dorsal.

**Variation.** Male: TL 16.0–19.1, PL 7.9–9.3, OL 8.1–9.8. Female: TL 18.6–24.4, PL 9.0–14.0, OL 9.6–12.8. Some females with lateral lobes bulging more medially and, thus, covering more strongly median septum (Fig. 3a). Posterior part of internal duct system relatively narrower and without strongly bulging parts (Fig. 3c).

#### Diagnosis

**Figure 4.**
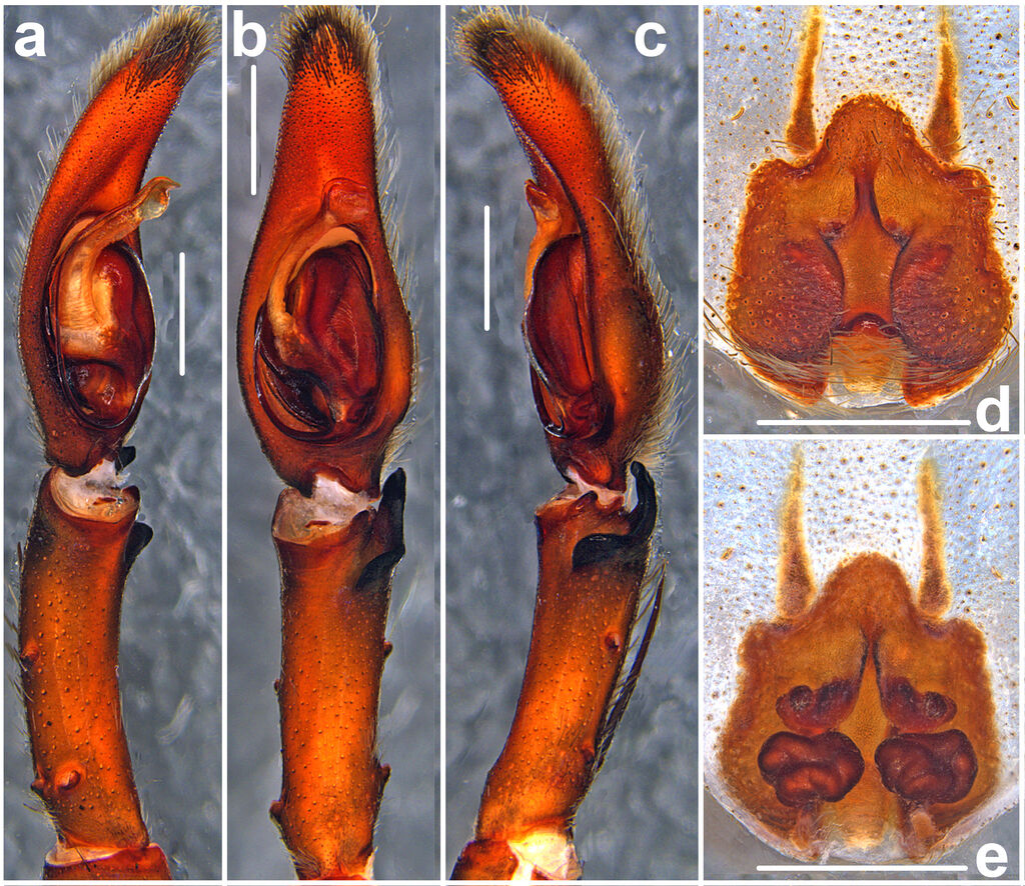
*Heteropodalebar* **sp. nov.**, male (**a**–**c**) and female (**d**–**e**) paratypes from Tanah Rata, Cameron Highlands, Pahang State, Malaysia, copulatory organs. **a**–**c** Left palp (**a** prolateral, **b** ventral, **c** retrolateral); **d** Epigyne, ventral; **e** Vulva, dorsal.

**Figure 5.**
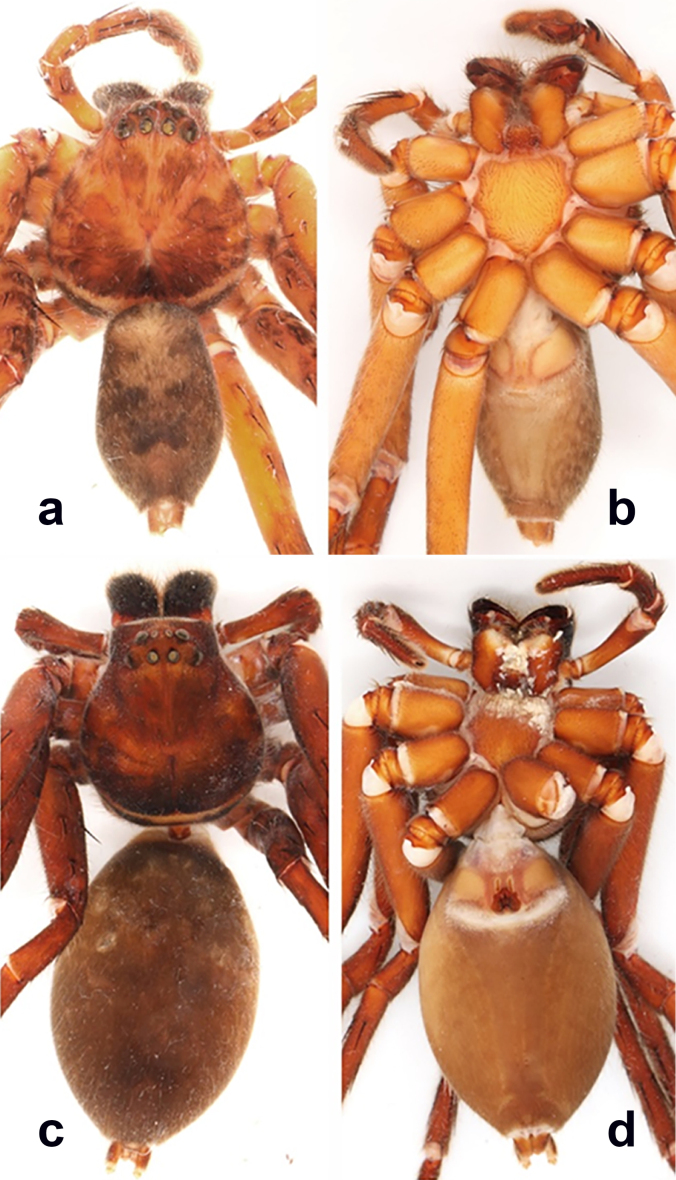
*Heteropodalebar* **sp. nov.**, Male habitus (**a** dorsal, **b** ventral) and female habitus (**c** dorsal, **d** ventral).

The males of *H.lebar* sp. nov. can be distinguished from those of all other congeners by the widened apical part of the conductor with retrolaterad, acuminate, triangular and rounded part (Figs 1, 4a–c). Females of *H.lebar* sp. nov. resemble *H.aemulans* Bayer & Jäger, 2009 (Bayer and Jäger 2009) by the shape of median septum in ventral view, but can be recognised by the following combination of characters (Figs 2, 3, 4d–e): 1. Width of anterior part of median septum one fifth of maximal width of median to posterior part, 2. Glandular appendages elongate, small rounded in ventral view, situated at lateral margin of first winding, 3. Posterior part of internal duct system wider than long.

#### Etymology

The specific name is derived from the Malay word lebar (ليبار), meaning broad and referring to the fact that the males with a widened conductor; adjective.

#### Distribution

**Figure 7.**
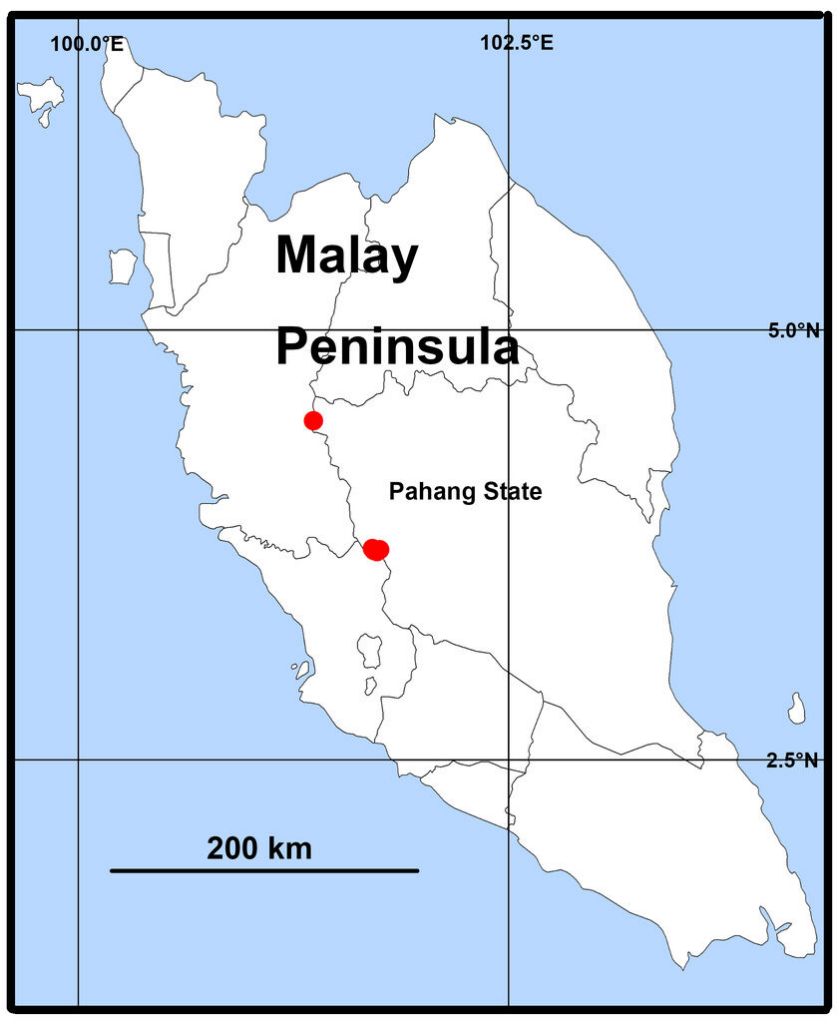
Distribution records of *Heteropodalebar* **sp. nov.**

Malaysia (Kuala Lumpur, Pahang State) (Fig. 7).

#### Biology

Spiders were observed during the night on the forest floor on the leaf litter of secondary or primary forests in elevations between 1000 and 1300 metres. Only in two females scars were found (one irregular elongated scar on left patella IV dorsally; one small round scar on right coxa III proximo-ventrally). It might be that mating bites do not often occur in this species.

## Discussion

This new species was previously identified as *H.tetrica* Thorell, 1897 (Thorell 1897) on the social network iNaturalist (https://www.inaturalist.org/home), especially the dark coloured females. Although there is a superficial resemblance of both species in having a strong sexual colour dimorphism with dark and more uniformly coloured females and lighter and vividly patterned males, there are distinct differences when looking in detail discerning both species even by interpreting photographs. *H.lebar* **sp. nov.** males have femoral spine patches fused in all legs (Fig. 6a), whereas these are separated at least in legs I–II in *H.tetrica* (Fig. 6c). Moreover, the dark pattern on the dorsal prosoma is more strongly developed in *H.lebar* **sp. nov.** (Fig. 6a), i.e. the large patches in the thoracic part reaching closer to the margin and are connected with marginal patches (vs. thoracic patches distinctly separated from margin in *H.tetrica*, Fig. 6c). Females of *H.lebar* **sp. nov.** have a uniformly dark colouration (Fig. 6b) with small light dots on opisthosoma and femora (vs. a variable, but generally lighter colour without such light dots in *H.tetrica*, Fig. 6d). However, for an unambiguous identification, dissection of the genitalia is recommended.

The internal duct system of females of *H.lebar* **sp. nov.** does not show any widened parts that could be easily discerned as spermathecae as, shown schematically for *Theridionmelanurum* Hahn, 1831 (Hahn 1831) and naturalistically for *Cupienniussalei* (Keyserling, 1877) (Keyserling 1877) (Foelix (2011): figs. 7.13a–b; Wiehle (1937): fig. 117 [sub *T.denticulatum* Walckenaer, 1805], 1967: fig. 29) (Walckenaer 1805, Wiehle 1937, Wiehle 1967, Foelix 2011). This scheme is widely used as the blueprint for female copulatory organs. Although schematically correct, this shape is rarely seen in the majority of spiders. Instead, there is rather a duct system as shown in Järvi (1912) and Järvi 1914 for Sparassidae (Bertkau 1872) that may or may not have any widened parts. In many *Heteropoda* species, these ducts of similar width are coiled and subsequently fused by sclerotisation of the surface of a spherical structure as shown, for example, in Jäger (2008: figs. 88, 92–93, 96, *H.hirsti* Jäger, 2008) (Jäger 2008). In this case, the ducts are kept as such. In other cases, the coiled ducts may fuse and build a secondarily widened receptacle (as in *H.christae*, Jäger, 2008: figs. 195, 197, 203) (Jäger 2008). In *H.lebar* **sp. nov.**, the stage of coiled ducts without fusion of the surface known from subadults (e.g. in *H.homstu* Jäger, 2008: fig. 271) (Jäger 2008) is apparently retained in the adult stage. That brings up the question of what can be called “spermathecae” in such a duct system. By definition, it should be the part in which sperm is deposited and maintained until being used for fertilising the eggs. As there is no morphological evidence of such a functional part, we avoid this (functional) term here. It might well be that, in all parts of the duct system behind the glandular pores, sperm is deposited as suggested by Jäger (2006: 60) (Jäger 2006). However, this needs to be confirmed through mating experiments, during which the specimens are frozen while copulating.

## Supplementary Material

XML Treatment for Heteropoda lebar

## Figures and Tables

**Figure 6a.**
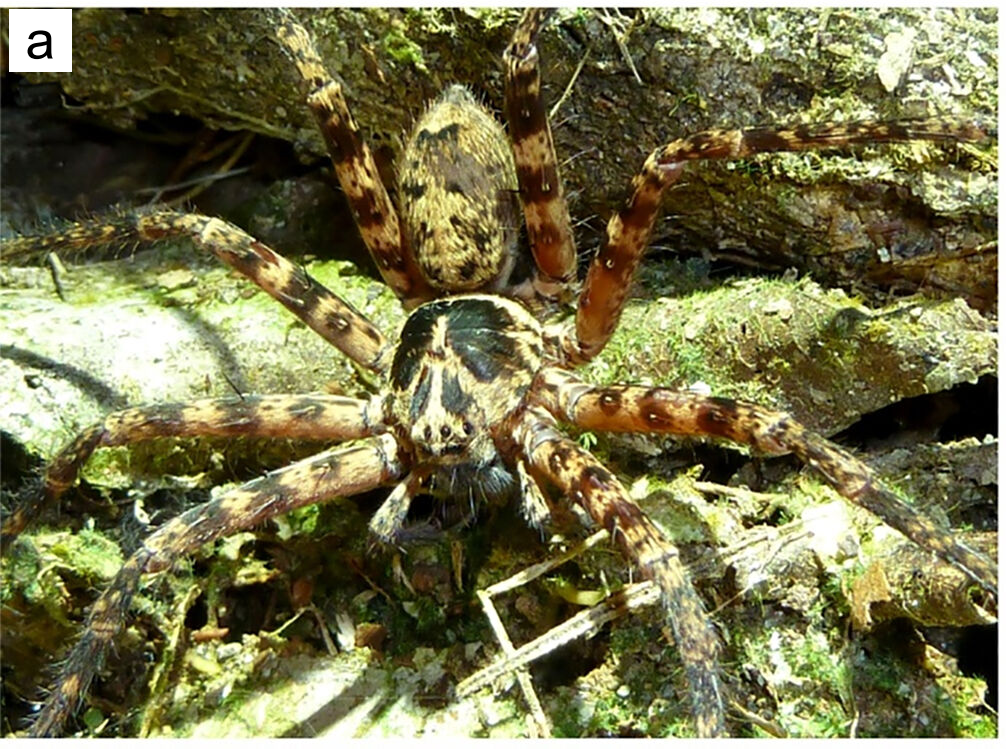
*H.lebar* **sp. nov.**, holotype male from Bukit Fraser, Pahang State, Malaysia;

**Figure 6b.**
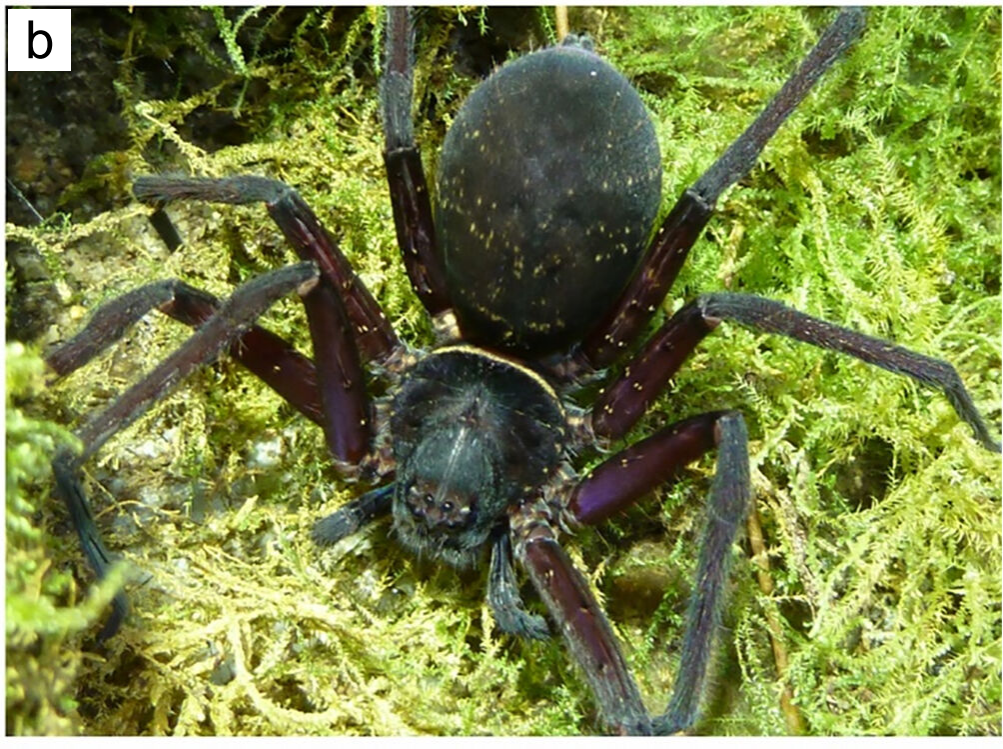
*H.lebar* **sp. nov.**, female paratype from Bukit Fraser, Pahang State, Malaysia;

**Figure 6c.**
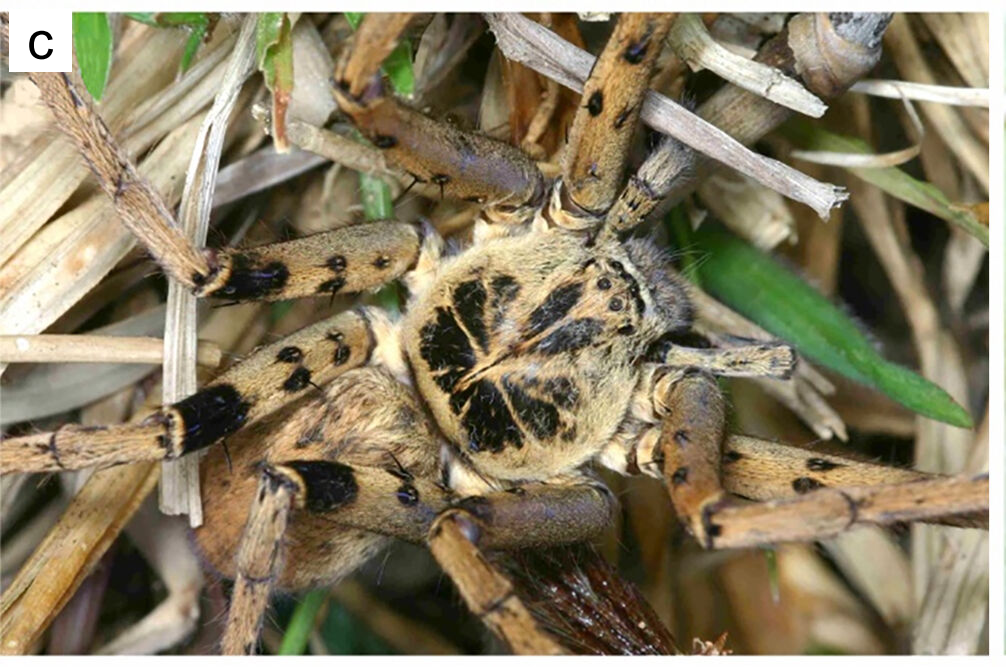
*H.tetrica* Thorell, 1897, male from Vang Vieng, Vientiane Province, Laos;

**Figure 6d.**
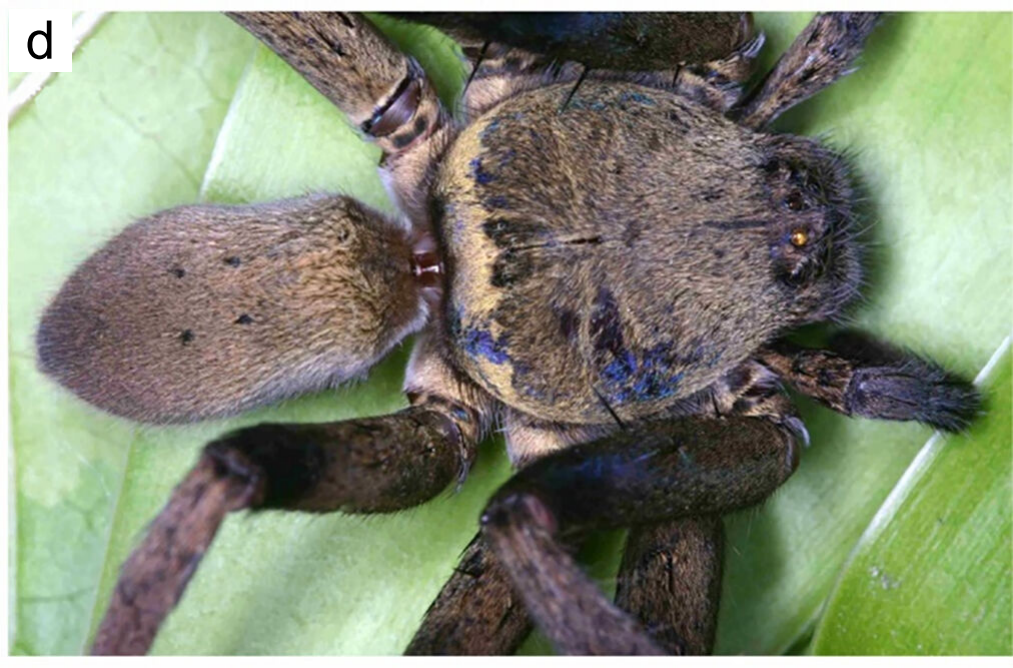
*H.tetrica* Thorell, 1897, female from Thakek, Kkammouane Province, Laos.
